# Diagnostic Performance for Differential Diagnosis of Atypical Parkinsonian Syndromes from Parkinson’s Disease Using Quantitative Indices of ^18^F-FP-CIT PET/CT

**DOI:** 10.3390/diagnostics12061402

**Published:** 2022-06-06

**Authors:** Miju Cheon, Seung Min Kim, Sang-Won Ha, Min Ju Kang, Hea-Eun Yang, Jang Yoo

**Affiliations:** 1 Department of Nuclear Medicine, Veterans Health Service Medical Center, Seoul 05368, Korea; jang8214.yoo@gmail.com; 2 Department of Neurology, Veterans Health Service Medical Center, Seoul 05368, Korea; neuroksm@bohun.or.kr (S.M.K.); vhsmcnr2005@bohun.or.kr (S.-W.H.); minju.kang@bohun.or.kr (M.J.K.); 3 Department of Physical Medicine and Rehabilitation, Veterans Health Service Medical Center, Seoul 05368, Korea; yanghe@bohun.or.kr

**Keywords:** atypical parkinsonian syndromes, Parkinson’s disease, dual-phase, ^18^F-FP-CIT PET, quantitative analysis

## Abstract

We are aimed to evaluate the diagnostic performances of quantitative indices obtained from dual-phase ^18^F-FP-CIT PET/CT for differential diagnosis of atypical parkinsonian syndromes (APS) from Parkinson’s disease (PD). We analyzed 172 subjects, including 105 non-Parkinsonism, 26 PD, 8 PSP, 1 CBD, 8 MSA-P, 9 MSA-C, and 15 DLB retrospectively. Two sequential PET/CT scans were acquired at 5 min and 3 h. We compared subregional binding potentials, putamen-to-caudate nucleus ratio of the binding potential, asymmetry index, and degree of washout. To differentiate APS, all BPs in both early and late phases (except late BPbrainstem) and all factors of the percent change except for putamen in APS significantly differed from PD. When a cut-off for early BPcerebellum was set as 0.79, the sensitivity, specificity (SP), positive predictive value (PPV), negative predictive value (NPV), and accuracy for differentiating APS 73.2%, 91.7%, 93.8%, 66.7%, and 80.0%. The early BPcerebellum showed significantly greater SP and PPV than the late quantitative indices. Combined criteria regarding both early and late indices exhibited only greater NPV. The quantitative indices showed high diagnostic performances in differentiating APS from PD. Our findings provide the dual-phase ^18^F-FP-CIT PET/CT would be useful for differentiating APS from PD.

## 1. Introduction

Parkinsonism is a neurological syndrome of the movement disorders characterized by bradykinesia, rigidity, tremor, postural instability, flexed posture, and freezing. There are many causes of Parkinsonism, with Parkinson’s disease (PD) reported as the most common. Atypical parkinsonian syndromes (APS), such as multiple system atrophy (MSA), progressive supranuclear palsy (PSP), corticobasal degeneration (CBD), and dementia with Lewy bodies (DLB), are other causes of Parkinsonism. Although the clinical progression and pathology can differ between Parkinsonism, there are evident difficulties for achieving confirmed diagnosis based solely on clinical features, particularly in the elderly and in patients at an early stage of disease [1,2]. To date, the therapeutic strategies for these chronic neurodegenerative disorders are based on symptomatic treatments. Monoamine oxidase inhibitor, dopamine agonists, levodopa formulations, and amantadine are well-known first antiparkinsonian drugs that are effective for symptom relief [3].

Accurate differential diagnoses of APS from PD are important for providing a prognosis, clarifying etiology and pathogenesis, and developing new therapeutic strategies. Functional brain imaging techniques were increasingly used in the diagnosis of Parkinsonism. Imaging of presynaptic dopamine transporters (DAT) availability is typically very sensitive for detecting Parkinsonism. However, DAT imaging is not effective for differentiating PD from APS [4]. A previous study showed a poor diagnostic result for ^123^I-beta-CIT SPECT to differentiate APS such as MSA, PSP, and CBD from PD [5]. Another study showed that the relative loss of the DAT did not significantly improve diagnostic accuracy in distinguishing between PD and MSA [6]. To overcome this limitation, various imaging tests were conducted to diagnose Parkinsonism differentially. Examples include ^18^F-FDG PET, simultaneous dual-radionuclide brain SPECT (^99m^Tc-ECD and ^123^I-FP-CIT) imaging, ^123^I-metaiodobenzylguanidine (MIBG) scintigraphy, and diffusion-weighted magnetic resonance imaging (MRI) [7,8,9,10,11]. Accurate diagnosis of PD and APS often requires several imaging tests. However, performance of two or more imaging tests can be inconvenient for patients, increases radiation exposure, and leads to unnecessary medical expenses.

^18^F-FP-CIT was synthesized as a dopamine transporter ligand for PET studies, and its pharmacokinetics was well established [12]. In studies of dynamic imaging, specific uptake in the striatum is reported to increase with a high first-pass influx rate (*K*_1_) evident in the first 15 min, indicates regional cerebral blood flow and peaks within 90–120 min in normal subjects. Several studies demonstrated that the level of functional activity in the structural and functional components of the central nervous system regulates the local rate of energy metabolism, and regional blood flow is adjusted based on local metabolic demand [13,14,15]. Dual-phase neuro PET imaging is a strategy used for other tracers based of the same initial high BBB extraction. Thus, we hypothesized that the diagnostic performance of differentiating PD and APS could be improved if we perform dual-phase ^18^F-FP-CIT PET/CT by adding the information on cerebral blood flow of early images, as compared to single-phase ^18^F-FP-CIT PET/CT (i.e., acquisition of only late static images at 3 h post-injection).

Although several studies showed the benefits of dual-phase ^18^F-FP-CIT PET/CT in diagnosing PD [16,17,18], only a few studies dealing with the dual-phase ^18^F-FP-CIT PET/CT to differentiate APS from PD [19,20]. In particular, there was no study suggesting the cut-off value through quantitative analysis. Our objective was to examine the clinical utility of quantitative factor in ^18^F-FP-CIT PET/CT imaging for differentiating between PD and APS.

Therefore, the purpose of this study was to retrospectively investigate whether quantitative analysis of dual-phase ^18^F-FP-CIT PET/CT is useful and to suggest a cut-off value for differentiating between PD and APS.

## 2. Materials and Methods

### 2.1. Subjects

This retrospective chart review study involving human participants was in accordance with the ethical standards of the institutional and national research committee and with the 1964 Helsinki Declaration and its later amendments or comparable ethical standards. The Human Investigation Committee (IRB) of Veterans Health Service Medical Center approved this study (IRB no. 2019-02-022, 2021-02-003). Informed consent from participants included in the study was obtained to publish the images. The database of all dual-phase ^18^F-FP-CIT PET/CT images ordered by three specialty-trained movement disorders neurologists (SWH, SMK, and MJK who had more than 10 years of experience with movement disorders) at the Veterans Health Service (VHS) Medical Center from November 2017 to February 2018 was reviewed. The images were commonly performed to differentiate PD from essential tremor, drug-induced parkinsonism, or other neurodegenerative diseases. The gold standard for diagnosis or differential diagnosis of PD is histopathology. Since this could not be performed in the current patients, clinical diagnostic standards were used to make a diagnosis. The assessment was performed by movement disorder specialists blinded to the results of the ^18^F-FP-CIT PET/CT. Clinical diagnosis was based on the current clinical diagnostic criteria. The UK Parkinson’s Disease Society Brain Bank Clinical Diagnostic Criteria for PD [21], the second edition of MSA diagnostic criteria developed by Gilman and collaborators in 2008 for MSA [22], the Clinical Diagnostic Criteria of the National Institute of Neurological Disease and Stroke and the Society for PSP [23], and revised criteria for the clinical diagnosis of probable and possible dementia with Lewy bodies for DLB [24] were used. All patients were assessed after at least 2-year of clinical follow-up. Patients with history of or concomitant major neuropsychiatric diseases, such as stroke, dementia, head trauma, hydrocephalus, and depression, were excluded. Brain CT or MRI was assessed to evaluate those conditions.

### 2.2. ^18^F-FP-CIT PET/CT

^18^F-FP-CIT PET (positron emission tomography)/CT (computed tomography) was performed using a Biograph 20 mCT PET/CT scanner (Siemens Medical Systems, Milwaukee, WI, USA). All patients had fasted for at least 6 h, and all drugs including antiparkinsonian drugs had been stopped 12 h before the scans. Two sequential PET and CT scans (dual-phase) were acquired at 5 min (early phase) and 3 h (late phase) after injecting 185 MBq (5 mCi) of ^18^F-FP-CIT. CT scanning began at the vertex and progressed to the skull base (30 mAs; 120 kVp; slice 3 mm) and PET imaging in the 3-dimensional (3D) mode immediately followed over the same region for a 10-min duration. The CT data were used for attenuation correction. The PET image was reconstructed using the OSEM (2 iterations, 21 subsets) and the TOF algorithm.

### 2.3. Image Analysis

**Figure 1:**
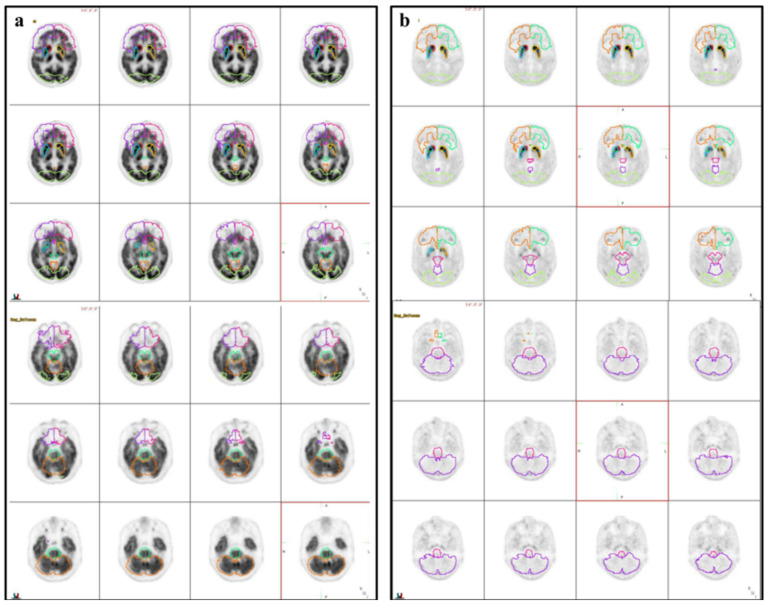
Illustration of volumes of interest (VOIs). Three-dimensional iso-contouring VOIs were automatically placed on the early (**a**) and late (**b**) phases of the ^18^F-FP-CIT PET/CT.

MIMneuro version 6.9.8 (MIM Software, Inc., Cleveland, OH, USA) was used for semi-quantitative volumetric analysis. A predefined three-dimensional volume-of-interest (VOI) was automatically placed for the quantification in the frontal lobe, brainstem, striatum, caudate, anterior putamen, posterior putamen, occipital lobe, and cerebellum both on early and late images. All 12 VOIs [six VOIs of bilateral striatal subregions (bilateral caudate nucleus, anterior putamen, and posterior putamen), bilateral frontal lobe VOI, one brainstem VOI, bilateral occipital VOI, and one cerebellar VOI] were drawn on respective PET (Figure 1). To compensate for anatomic variation the fitting algorithm includes an automated adjustment of the VOI’s. Quantification indices of VOIs were defined as follows. First, the binding potential (BPs) was calculated from the mean values of the VOIs using the cerebellum as a reference region, resulting in these BPs (BPstriatum, BPcaudate, BPputamen, BPanterior putamen, BPposterior putamen, BPfrontal lobe, BPbrainstem, BPcerebellum, and BPoccipital lobe). The BPs was defined as follows: (Average of two sides of the SUVmean of the subregional VOI–the SUVmean of the whole brain VOI)/SUVmean of the whole brain VOI. Second, the putamen-to-caudate nucleus ratio of the BP (BPcaudate/BPputamen) was calculated to analyze the relationship of DAT availability between those two regions. Third, the asymmetry between the right and the left sides of the putamen was evaluated using an asymmetry index (AI), which was calculated by [(better side BP–worse side BP)/average BP of the two sides] × 100 (%). Finally, the degree of washout was defined as follows: (SUVmean of VOI on 3 h image-SUVmean of VOI on 5 min image)/SUVmean of VOI on 5 min image. Images were interpreted by experienced nuclear medicine physicians (MC, JY) who were completely unaware of the clinical information analyzed all images. Indices were calculated and evaluated the group difference.

### 2.4. Statistical Analysis

Statistical analyses were performed using SPSS version 18.0 software for Windows (SPSS Inc., Chicago, IL, USA). Descriptive statistics were reported as means and standard deviations, and continuous variables were reported as medians and standard deviation. Categorical variables were reported as frequencies and percentages. Comparisons of values between groups were performed using the independent samples *t*-test for continuous variables and the Chi-square test for dichotomous variables. Differences in diagnostic efficacy of each significant quantitative index for differentiation between PD and APS were compared using McNemar’s test. Receiver operating characteristics (ROC) curve analysis was performed to measure the accuracy of cut-off values of significant criterion to differentiate PD from APS. The diagnostic accuracy was expressed as the area under the corresponding ROC curve (AUC). The corresponding sensitivity (SN), specificity (SP), positive predictive value (PPV), negative predictive value (NPV), and accuracy (AC) were calculated for each finding. A P value of less than 0.05 was considered to indicate a statistically significant difference for all analyses.

## 3. Results

Two hundred eight subjects who had undergone dual-phase ^18^F-FP-CIT PET/CT were included. Eighteen subjects with stroke, 8 subjects with dementia, 6 subjects with concomitant depression, 3 subjects with hydrocephalus, and 1 subject with a history of head trauma were excluded. Finally, 172 subjects were analyzed. Of the 172 subjects, 154 were men and 18 were women, with a mean age of 74.15 ± 6.15. One hundred five non-parkinsonism, 26 PD, 8 PSP, 1 CBD, 8 MSA-parkinsonian type (MSA-P), 9 MSA-cerebellar type (MSA-C), and 15 DLB cases were analyzed (Table 1). In our study, the incidence of Parkinsonism was 38.95% (67/172), PD was 15.11% (26/172), and APS was 23.84% (41/172). The remaining subjects were proven as non-Parkinsonism, such as essential tremor, drug-induced Parkinsonism, vertigo, primary dystonia, or peripheral neuropathy. The mean duration of disease in the patient group with Parkinsonism was 47.92 ± 44.04 months.

**Figure 2:**
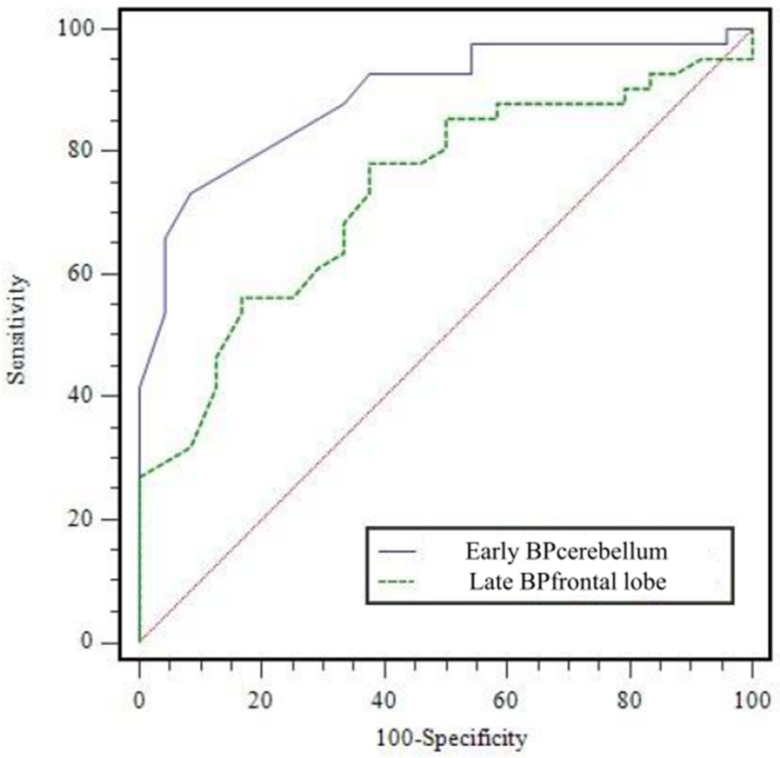
Receiver operating characteristic curve of early BPcerebellum and late BPfrontal lobe for diagnosing atypical Parkinsonian syndromes. AUC was 0.890 ± 0.040 (*p* < 0.0001) for early BPcerebellum and 0.734 ± 0.063 (*p* = 0.0002) for late BPfrontal lobe. AUC = area under curve.

All BPs in both early and late phases (except late BPbrainstem) in APS significantly differed from PD (all, *p* < 0.05). However, there were no significant differences in AI index and BPcaudate/BPputamen for either the early or late phase (Table 2). ROC analysis for differentiation between APS and PD, the early BPcerebellum showed the highest AUC (0.890 ± 0.040, *p* < 0.0001, Figure 2). When a cut-off for early BPcerebellum was set as 0.79, the sensitivity, specificity, positive predictive value (PPV), negative predictive value (NPV), and accuracy for differentiating APS were 73.2% (30/41), 91.7% (22/24), 93.8% (30/32), 66.7% (22/33), and 80.0% (52/65), respectively. It is a significantly higher AUC value than that of the late BPfrontal lobe (AUC = 0.734 ± 0.063, *p* = 0.0002), which is the best quantitative factor among the late parameters. In addition, the early BPcerebellum showed significantly greater SP and PPV than the late BPfrontal lobe. The diagnostic performance of the index with the highest AUC value among the early index, the index with the highest AUC value among the late index, and the index combining the two (low early BPcerebellum ≤ 0.79 and low late BPfrontal lobe ≤ 1.25) for differentiating APS are presented Table 3. After comparison analysis, combined criteria exhibited only greater NPV than early BPcerebellum (*p* = 0.1466), and no significant differences in SN, SP, PPV, and AC were observed.

**Figure 3:**
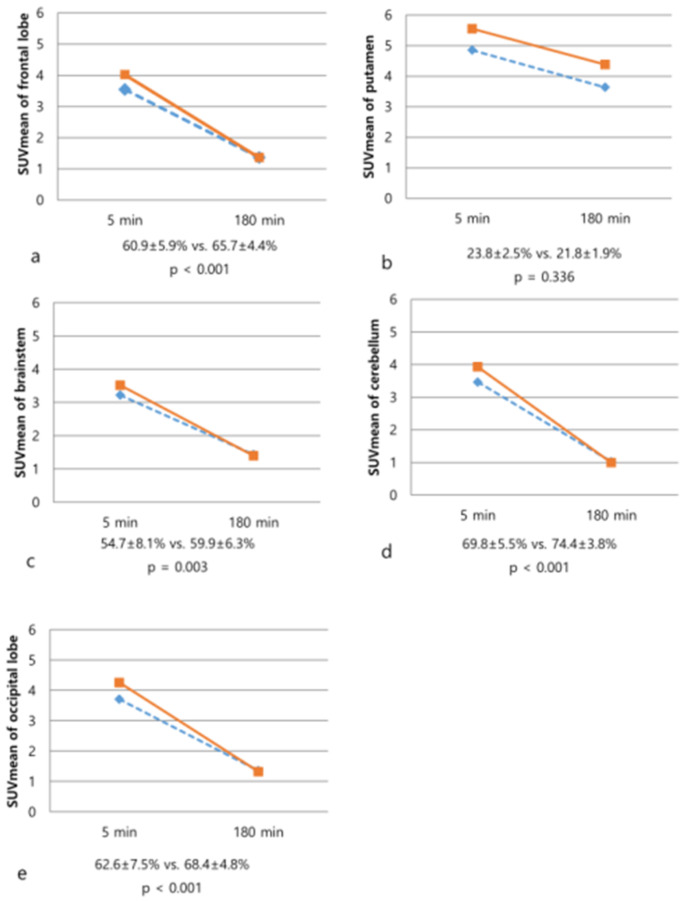
Changes of SUVmean values on 5-min and 180-min images of ^18^F-FP-CIT PET/CT in the patients with atypical Parkinsonian syndromes (dotted line) and Parkinson’s disease (solid line). The degree of washout of frontal lobe (**a**), brainstem (**c**), cerebellum (**d**), and occipital lobe (**e**) was significantly increased in the PD. In contrast, there was no significant difference in the degree of washout of the putamen (**b**, *p* = 0.336).

Percent change factors in differentiation between APS and PD, all factors of the percent change except for putamen were statistically significant (all, *p* < 0.05). In the case of APS, we can confirm that the increased rate of BP between early and late phases in the extrastriatal region was higher than that of the PD. On the other hand, the increased rate of BP of striatal region was relatively higher in the PD. In other words, in the case of APS, the BP of the extrastriatal region showed a difference from the PD in the early phase, but no such difference was seen in the late phase (Figure 3).

**Figure 4:**
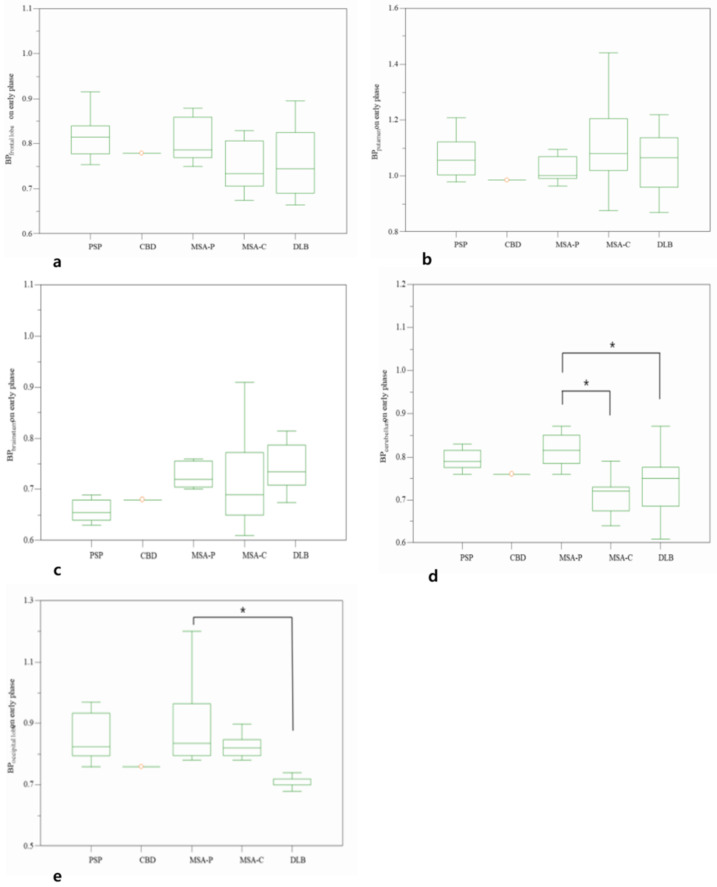
Comparative BP values on early phase of frontal lobe (**a**), putamen (**b**), brainstem (**c**), cerebellum (**d**), and occipital lobe (**e**) among subtypes of atypical parkinsonian syndromes. * *p* < 0.05.

In addition, a comparative analysis was performed for subtypes in the APS. Early BPcerebellum of MSA-C (0.71 ± 0.04) and DLB (0.73 ± 0.06) were significantly low from that of MSA-P (0.85 ± 0.13). Early BPoccipital lobe of DLB (0.72 ± 0.05) was significantly different from that of MSA-P (0.90 ± 0.14). However, there was no significant difference in the BPfrontal lobe (*p* = 0.365), BPputamen (*p* = 0.501), and BPbrainstem (*p* = 0.168) between subgroups of the APS (Figure 4).

**Figure 5:**
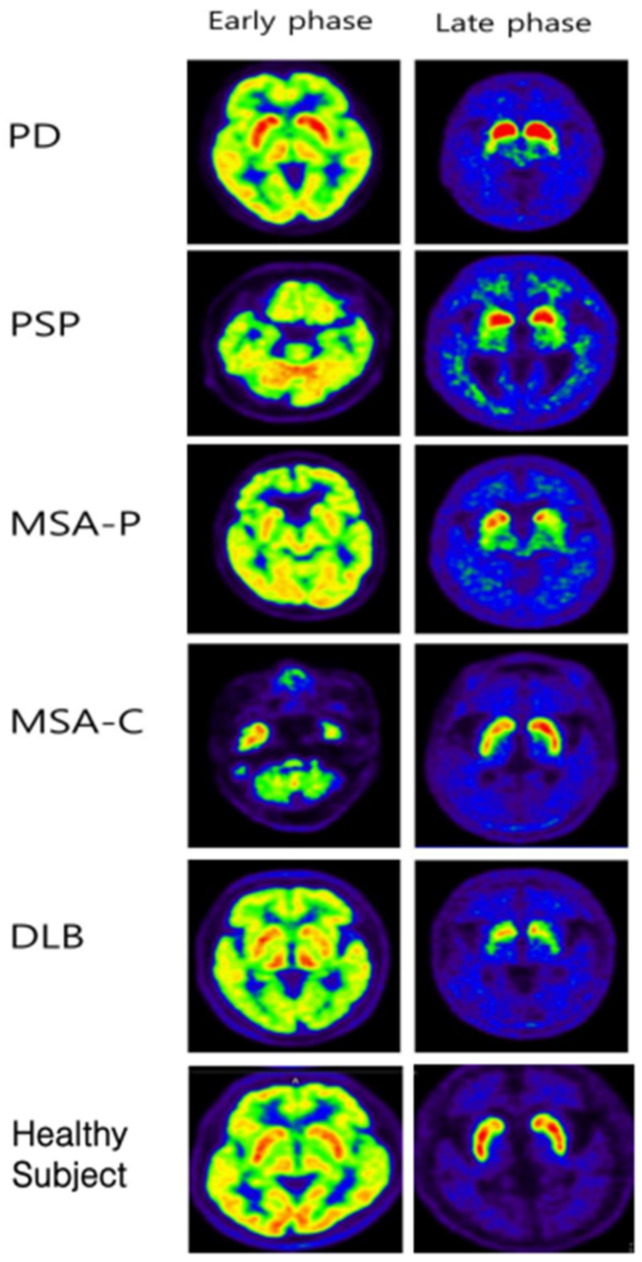
Representative images of early and late phases ^18^F-FP-CIT PET/CT from PD, PSP, MSA-P, MSA-C, and DLB patients. In the late phase, even in APDs such as DLB, a rostocaudal gradient was observed, and in MSA-C patients, the asymmetric index was increased. In other words, it can be said that it is difficult to differentiate between PD and APS using only the late phase image.

For differentiation between Parkinsonism and non-Parkinsonism, all BP of striatal subregions and the BPcaudate/BPputamen in both the early and late phases significantly differed (all, *p* < 0.05). In contrast with the BPs of striatal subregions, BPs of the extrastriatal regions were not significantly different between Parkinsonism and non-Parkinsonism. For the AI, only the late phase striatum (*p* < 0.001) and putamen (*p* < 0.001) showed significant differences. Representative cases are illustrated in Figure 5.

## 4. Discussion

Differentiation of Parkinsonism into PD or APS in everyday clinical practice is challenging. Accurate differential diagnosis of APS has cardinal therapeutic and prognostic importance. Since no effective treatment was developed for APS, and the prognosis of patients with APS is poorer than that of patients with PD. In the early stages of Parkinsonism, it is often difficult to know whether a patient’s disease is due to PD or another condition that mimics it. Approximately 10 to 25% of patients are misdiagnosed even by movement disorder specialists [25,26]. Several different procedures such as cardiac MIBG scintigraphy, transcranial ultrasound, functional magnetic resonance imaging, FDG-PET were applied to improve diagnostic accuracy [27]. A few studies using ^18^F-FP-CIT PET/CT in APS suggested that it could be helpful in the differential diagnosis of APS. Overall, it is known that most of Parkinsonism has more severe dopaminergic neuron degeneration in one striatum in the early stage, leading to asymmetric dopaminergic reduction. In addition, it is known that PD shows a specific pattern of uptake patterns in the striatum called “rostrocaudal gradient”, which is the first to decrease dopamine transporter in the posterior putamen. The caudate nucleus shows the sequential change, so the uptake ratio of putamen/caudate in the striatum decreases [26,28,29,30]. Despite the previous results, differential diagnosis of APS using striatal DAT loss alone remains the challenge, and no studies have suggested a cut-off value that distinguishes APS and PD. We observed that striatal DAT binding significantly decreased in patients with Parkinsonism compared to patients with non-Parkinsonism. To determine whether a patient with Parkinsonism, standard late phase ^18^F-FP-CIT PET/CT obtained 3 h after injection was sufficient to acquire excellent diagnostic results. On the standard late phase images, we confirmed striatal DAT loss in both PD and APS. However, to discriminate between APS and PD, it was difficult to obtain sufficient diagnostic results only with late phase ^18^F-FP-CIT PET/CT imaging, and there was no significant difference in the asymmetric index and the putamen-to-caudate nucleus ratio between APS and PD (*p* > 0.05). The best quantitative index in the late phase exhibited poor specificity, PPV, and accuracy. These results are consistent with those of Park et al. [18], who showed no significant difference in the DAT reduction patterns of patients with PD and APS. However, in that study, there was no further study on how to differentiate it. We identified it is possible to reinforce the diagnostic performance in differentiation between PD and APS with additionally obtaining the early phase. Moreover, we presented the cut-off value through quantitative analysis and showed high diagnostic results when discriminating APS using each cut-off value in the early and late phases. Among several quantitative indices, the early BPcerebellum exhibited the highest diagnostic performance. In particular, the specificity and PPV were significantly higher than those of the late quantitative indices. Although there was a significant difference in diagnostic performance between the early BPcerebellum and combined criteria (low early BPcerebellum ≤ 0.79 and low late BPfrontal lobe ≤ 1.25) only in NPV, this may be due to the small number of subjects. In differentiating APS from PD, a statically significantly higher diagnostic performance was obtained when the quantitative factors on the early phase were considered together, rather than when only the quantitative factors obtained from the late phase were considered. Therefore, if dual-phase ^18^F-FP-CIT PET/CT is performed, we cannot only diagnose Parkinsonism, but also differentiate between APS and PD.

In the presentedstudy, we identified significantly early peak striatal ^18^F-FP-CIT uptake in patients with PD. These results are consistent with previous studies that reported shortened equilibrium state in PD patients [6,14,31,32]. In addition to this, our study further revealed different peaks for extrastriatal ^18^F-FP-CIT uptake and different washout between PD and APS. There is later peak activity, and slighter decreases in the extrastriatal regions such as frontal lobe, brainstem, occipital lobe, and cerebellum in patients with APS compared to that in patients with PD. Having different washouts such as this may result from DAT availability, serotonin receptor density, peripheral tracer clearance, and regional cerebral blood flow. There were no significant differences in all quantitative indices between the subgroups of APS because it may be due to too small subject to obtain meaningful results. However, in some subtypes, we can confirm that the early BPoccipital lobe and early BPcerebellum showed significant differences from other subgroups. These findings generally correspond with several previous studies that identified glucose metabolism patterns with PD, MSA, PSP, and DLB [33]. Therefore, considering quantitative indices of ^18^F-FP-CIT uptake in the early phase, it may be possible to suggest which subgroup it corresponds to within APS.

There are some limitations to our study. First, relatively small number of APS subjects was included in the study. This can bias the results on both standard late phase and early phase have shown differential impacts of the distinct APS on these imaging metrics. Second, this is a retrospective single-center study, and not all subjects underwent dual-phase ^18^F-FP-CIT PET/CT for differentiating APS from PD, which may induce selection bias. Third, there was no pathologic confirmation of the clinical diagnosis and the follow-up period of patients was relatively short. Therefore, misdiagnosis could have happened because of the lack of postmortem verification. However, at least two years, the follow-up period was sufficient for our experienced neurologists to establish a diagnosis in our study. Fourth, there are far more men than women in the study. The gender ratio imbalance of the study subject may have influenced the cut-off value in the quantitative analysis since the DAT availability is known to be higher in women than men. Fifth, we set the whole brain as a reference region for quantitative analysis. This is a widely used method for quantitation in neurodegenerative diseases [34]. However, there is no appropriate reference region for every parkinsonian group, decreased uptake of occipital lobe in advanced PD and DLB and cerebellum in MSA-C. Therefore, further prospective studies with larger numbers of participants are warranted to investigate the diagnostic value of our suggested ^18^F-FP-CIT PET/CT criteria for APS.

The data indicate it seems likely that early phase imaging will give additional value for differentiating APS from PD. Moreover, quantitative assessments are effective in interpreting ^18^F-FP-CIT PET/CT, thereby leading to more accurate diagnoses for APS. The use of combined information from early and late phase ^18^F-FP-CIT PET/CT images is a promising and clinically relevant approach for improving the differential diagnosis of Parkinsonism. Therefore, the application of dual-phase ^18^F-FP-CIT PET/CT using quantitative analysis would be useful for differentiating APS from PD.

## Figures and Tables

**Table 1:** One hundred seventy-two subject’s characteristics were expressed as mean ± standard deviation. Data represented are mean values for continuous variables and proportions for categorical variables.

	Non-Parkinsonism (n = 105)	PD (n = 26)	PSP (n = 8)	CBD (n = 1)	MSA-P (n = 8)	MSA-C (n = 9)	DLB (n = 15)
Age (years)	74.2 ± 6.2	74.1 ± 6.1	74.5 ± 5.9	72	74.3 ± 6.3	74.3 ± 5.6	74.6 ± 6.3
Gender (M/F)	100/5	21/5	7/1	1/0	6/2	9/0	15/0
Disease duration (months)	47.7 ± 44.3	47.5 ± 44.1	48.4 ± 44.9	3	49.1 ± 45.0	43.8 ± 42.9	49.3 ± 45.7
MMSE score	23.1 ± 4.7	23.1 ± 4.7	23.3 ± 4.5	21	23.1 ± 4.8	23.6 ± 4.3	22.8 ± 5.0
GDS score	2.7 ± 1.3	2.7 ± 1.3	2.8 ± 1.2	3	2.8 ± 1.3	2.7 ± 1.2	2.9 ± 1.2

**Table 2:** Summary of quantitative indices for differentiation between atypical Parkinsonian syndromes and Parkinson’s disease. Data represented are mean ± standard deviation values. NS = not significant.

Quantitative Indiceson Early Phase (5 min)	APS (n = 41)	PD (n = 26)	*p* Value	Quantitative Indiceson Late Phase (3 h)	APS (n = 41)	PD (n = 26)	*p* Value
**BPstriatum**	1.08 ± 0.19	1.32 ± 0.20	<0.001	BPstriatum	3.59 ± 0.96	4.58 ± 1.45	0.001
**BPcaudate**	1.03 ± 0.18	3.78 ± 12.83	<0.001	BPcaudate	4.12 ± 1.01	5.15 ± 1.60	0.003
**BPputamen**	1.19 ± 0.23	1.27 ± 0.29	<0.001	BPputamen	3.25 ± 0.94	4.214 ± 1.43	0.002
**BPanterior** **putamen**	1.16 ± 0.20	1.41 ± 0.21	<0.001	BPanterior putamen	3.85 ± 1.09	5.03 ± 1.66	0.001
**BPposterior** **putamen**	1.03 ± 0.19	1.27 ± 0.23	<0.001	BPposterior putamen	2.65 ± 0.82	3.38 ± 1.29	0.001
**BPfrontal lobe**	0.92 ± 0.10	0.80 ± 0.13	<0.001	BPfrontal lobe	1.28 ± 0.09	1.21 ± 0.14	<0.001
**BPbrainstem**	0.80 ± 0.12	0.71 ± 0.08	<0.001	BPbrainstem	1.30 ± 0.10	1.26 ± 0.11	NS
**BPcerebellum**	0.89 ± 0.11	0.76 ± 0.09	<0.001	BPcerebellum	0.93 ± 0.05	0.90 ± 0.12	0.018
**BPoccipital lobe**	0.97 ± 0.13	0.83 ± 0.17	<0.001	BPoccipital lobe	1.25 ± 0.08	1.20 ± 0.15	0.017
**Striatum** **asymmetry**	2.611 ± 2.44	1.95 ± 1.62	NS	Striatum asymmetry	9.76 ± 0.43	9.43 ± 10.72	NS
**Caudate** **asymmetry**	5.74 ± 0.50	3.09 ± 2.60	NS	Caudate asymmetry	8.63 ± 0.63	8.91 ± 1.43	NS
**Putamen** **asymmetry**	2.41 ± 1.96	2.70 ± 2.37	NS	Putamen asymmetry	3.25 ± 0.94	4.214 ± 1.43	NS
**BPcaudate/BPputamen**	1.21 ± 0.97	1.06 ± 0.08	NS	BPcaudate/BPputamen	0.78 ± 0.08	0.82 ± 0.12	NS

**Table 3:** Comparisons of diagnostic performance between quantitative indices for differentiating APS. Combined criteria means Late BP_frontal lobe_ ≤ 1.25 and Early BP_cerebellum_ ≤ 0.79. * ^†^ *p* < 0.001, ^‡^ *p* < 0.05 using McNemar test. NPV = negative predictive value; PPV = positive predictive value.

Diagnostic Criteria	Sensitivity (%)	Specificity (%)	PPV (%)	NPV (%)	Accuracy (%)
**Late BP_frontal lobe_** **≤ 1.25**	73.17 (30/41)	54.17 * (13/24)	73.17 * ^‡^ (30/41)	54.17 * ^†^ (13/24)	66.15 (43/65)
**Early BP_cerebellum_** **≤ 0.79**	73.17 (30/41)	91.67 * ^†^ (22/24)	93.75 ^‡^ (30/32)	66.67 ^†^ (22/33)	80.00 (52/65)
**Combined criteria**	60.98 (25/41)	95.83 ^†^ (23/24)	96.15 * (25/26)	95.83 * (23/24)	73.85 (48/65)

## Data Availability

The data that support the findings of this study are available from the corresponding author M.C., upon reasonable request.
